# Remarks on Sepsis and Its Treatment

**Published:** 1902-03

**Authors:** James E. King

**Affiliations:** Buffalo, N. Y.; Lecturer in Embryology; instructor in obstetrics, University of Buffalo; assistant attending gynecologist, Buffalo General Hospital; assistant obstetrician, Erie County Hospital; Fellow of the British Gynecological Society; member New York State Medical Association, etc.


### BUFFALO MEDICAL JOURNAL.

VOL. XLI.—LVII. MARCH, 1902. No. 8.

### ORIGINAL COMMUNICATIONS.

### Remarks on Sepsis and Its Treatment.

BY JAMES E. KING, M. D., Buffalo, N. Y.

Lecturer in Embryology; instructor in obstetrics, University of Buffalo; assistant attending gynecologist, Buffalo General Hospital; assistant obstetrician, Erie County Hospital; Fellow of the British Gynecological Society; member New York State Medical Association, etc.

THE recent advances in bacteriology have brought the germ theory of disease into the domain of science. Except a very few of the infectious processes, the specific cause has been definitely determined and described and prophylaxis and preventive medicine form a very great part of our treatment. In no way has this knowledge been of greater service than in our understanding of sepsis. By prophylaxis and antiseptic treatment, the hundreds of women who yearly succumbed to puerperal fever and those who died of septic wounds have been reduced to inconsiderable numbers. But while our knowledge has been of inestimable value in reducing the frequency, yet with but few exceptions has it enabled us to cut short an infectious disease by direct attack upon the germ or their toxins. Diphtheria, tetanus, and rabies may claim a specific treatment, based upon our knowledge of the causes, but the vast majority still run their course, modified only by our efforts. This is true in obscure cases of sepsis, and puerperal fever, which is well developed, is as alarming to the practitioner of today as in earlier times when our knowledge was much less. It is my purpose to consider sepsis briefly, and to determine if as yet we have a treatment for these conditions.

Under the general term of sepsis, I include those conditions which arise as the result of the activity of the saprophytic or the pyogenic cocci. The conditions thus arising may be classi-

1. Read before the Roswell Park Medical Club, January 6, 1902.

fied under the headings of sapremia, or septic intoxication, and septicemia, or septic infection. The bacteria causing sapremia are of interest to us only in the part they play in mixed infections by their association with the pyogens. In sapremia the bacteria are acting upon dead organic matter and it is the absorption of the ptomaines alone that produces the familiar clinical picture. The bacteria are at work outside of the living tissues and constitutional treatment then could have no effect or in any way influence the growth of the germs. As the causative factor in septicemia, the pyogens occupy the first rank, and it is seldom that we see sepsis arising alone from the colon bacillus or the gonococcus, although these are not infrequently seen as mixed infections. In septicemia the bacteria work on living tissues, resulting in the destruction of the cells and the production of leucomaines which are thus readily absorbed. This is the simplest expression of septic infection which may go a step further and the germs themselves enter the blood and exert their influence on the blood as a tissue. Or the process may even proceed further and bits of the suppurating focus may enter the veins and be carried to remote parts of the body, resulting in pyemia. These are the three expressions of septic infection, all due to the pyogens and their products.

Let us look for a moment at the characteristics and behavior of these pus germs in the tissues. The true pus producers are classified as the staphylococcus and the streptococcus. The former are socalled because of their appearance in clusters under the microscope. They are durable, being quite resistant to the action of antiseptics and requiring several minutes boiling for their destruction. In the tissues they are usually found in circumscribed collections of pus, and they do not tend to spread, as around such a process is found a barrier of round cell infiltration which tends to its limitation. These germs are the principal ones in furunculosis, abscesses, and empyemas. The strepcococci are seen in chains and they also are resistant to heat and germicides, but they differ in one particular from the staphylococci, in that they tend to spread through the medium of the lymphatics, and we find them concerned in the production of cellulitis, peritonitis, and all spreading inflammations. These two varieties are commonly associated. Besides these, however, in situations convenient for the infection, the colon bacillus is often found. The same is true of the gonococcus, although the latter is sometimes found alone. Thus we see that in septicemia the bacteria are exerting their activity on living tissue, and it is quite possible that treatment through the general circulation and lymphatics may be expected to find its way to the focus of absorption, and to influence the germs and toxins in the blood. Clinically, we find a great difference in the behavior of germs which under the microscope and in growth are exactly the same. The one may be extremely virulent and the other toxic to a comparatively mild degree. This is especially true of the streptococcus and also a well-known fact of the Klebs-Löffler bacillus. So far this phenomenon has been explained by theory only and facts are still wanting.

The morbid changes, as the result of infection vary greatly, depending upon this same virulence, and the length of time that the process has been in progress. The blood is naturally the first tissue of the organism to suffer, and the well known changes in the white cells are produced and the red cells and their carrying power much reduced. Cocci are often found in the blood and the toxins are also found in the fluid element. The changes found in other tissues are due in part to the impoverishment of the blood and also to the toxins. The liver and kidneys as organs of elimination are the first to suffer, and cloudy swelling is usually the result. The heart also receives its share, and the same changes are found.

From the knowledge, then, we have of sepsis, the indications for treatment are quite clear; first, to be rid of the focus of trouble and to prevent further development, and second to neutralise the toxins and their effects. To meet these indications we naturally turn to the serum treatment. Here, however, we are doomed to disappointment, for the antitoxin serum for sepsis has been an almost unqualified failure. Early in the '90's Marmarek prepared a serum that was fatal to rabbits in extremely small dosage. With this he obtained the antistreptococcus serum, and in a large number of cases in which he used it, he met with great success. Great hopes were entertained that at last a specific for sepsis had been found, but subsequent investigators failed to verify Marmarek's flattering results. In 1900, the French themselves declared the serum a failure, and that its use was being abandoned. American surgeons also declared against it and the report of a committee from the American Gynecological Society, appointed for the purpose of deciding its merits, reported unfavorably. The reports of Marmarek were based upon the use of the serum in a large number of cases of erysipelas, and as this disease tends to self limitation and improvement in from seven to ten days, it leads one to think that the improvement took place uninfluenced by treatment. The causes for the failure have been attributed to the use of poor serum, delay in treatment and insufficient dosage. But the best theoretical reason is based upon the fact that, clinically, infections are almost always mixed and that in administering the antistreptococcus serum we are combating but one germ and its effect, and the others are left to disport themselves with impunity in the tissues. So we must relegate antistreptococcus serum with tuberculin to the past and admit that today we have no successful serum treatment for sepsis. The use of antiseptics for attacking the bacteria in their lair through the circulation has been proposed and tried, and the intravenous injection of bichloride and subcutaneous use of carbolic acid have had their day, as the results in no way encouraged or justified their further trial.

There is one treatment that has been before the profession for some years and the reports from which justify our attention. I refer to the silver treatment of Credé. My own interest in the method was stimulated by a number of visits this past summer to Credé, and opportunity afforded to see the application of the remedy by the originator himself. The reports from this treatment are more encouraging than those of any other before proposed, and silver possesses the decided advantage of being nonpoisonous in any dosage. Clinically, it has long been observed that when silver wire was used as suture material suppuration never occurred around the wire, while other sutures but little removed suppurated, perhaps for their entire tract. Laboratory experiment confirmed these facts and to silver we must accredit some peculiar and unexplained property that at least inhibits the growth of bacteria.

This knowledge led to many attempts to utilise this property of silver and catgut by silver nitrate was not unknown. It was left for Credé, however, to make the application of silver and its salts practical. The first attempts were in the use of two salts of silver—the citrate and the lactate. The citrate, or as known by its trade name, itrol, is a stable, nonpoisonous white powder, soluble in 1-4000. Its principal use is as a dusting powder, and as such it as given the greatest satisfaction, but whether or not it possesses any marked advantage over the many excellent dusting powders we now have, the writer is unable to state. It may also be used in the proportion of its solubility as an irrigating fluid. The lactate or actol is of considerably greater interest. This also is a white powder, soluble in 1-25. As a solid it is somewhat irritating to the tissues; as a solution, however, it possesses all the antiseptic and germicidal properties of the bichloride without its disadvantages. In strong solutions it may be used for irrigating the bowels and uterus without fear of poisonous absorption. It is claimed that the silver salt has the same germicidal action as bichloride when used in double the proportion. From this salt of silver are prepared sponges, catgut and gauze.

An attempt was made to use these salts for their constitutional effect in sepsis and experimentation with animals seemed to promise much, but it was soon found that these salts in solutions of sufficient strength quickly combined with the albumins and were rendered inert. It was therefore seen that to use silver internally, a preparation must be found that was soluble in albumins and would circulate in its metallic form. Efforts in this direction brought forth an allotropic form of silver, known as collargolum, which answers the requirements. It is a pure silver in allotropic form, possessing markedly different properties from the form of silver with which we are familiar. Collargolum is soluble in water in proportion of 1-25, in the weaker solutions, being more readily soluble. There may be at the bottom of the container, however, even with the greatest care, a small amount of insoluble silver. In using solutions for intravenous injection this fact should be remembered and the perfect solution poured off for use. For whatever purpose the solutions should be freshly made, and only distilled water should be used in its preparation; it should be protected from the light, although as a peculiar fact, solutions with the addition of 1 per cent. of albumin are rendered much more stable and they may be kept for a considerable time without deteriorating. Heating to a temperature of 176° F. changes all solutions by a precipitation of insoluble silver. For irrigation it may be used in strengths of from 1-10,000 to 1-100 in septic wounds and cavities. For intravenous injection 5 to 15 grains may be used in a ½ per cent. solution, using only the perfect solution as suggested above. The reaction that follows is no more than that following the introduction of any fluid directly into the circulation. By mouth it may be given in solution or in pill form, but all solutions should contain a percentage of albumin, otherwise the acid of the stomach renders it insoluble.

The most available form, however, is the ointment or unguentum Credé, which contains 15 per cent. of collargolum. It should be protected from the air and light to prevent deterioration. It is used by inunction in doses of 15-45 grains, every 4, 6, or 24 hours, according to the requirements of the case. The technique of its application is of importance and in order to obtain the best results the usual sites for inunction are thoroughly cleansed with soap, water and brush and 20-30 minutes devoted to applying the ointment; the surface is then covered with a piece of protective to insure better absorption. The silver is taken up by the general circulation and can be easily demonstrated in the skin and deeper tissues at the site of the inunction.

The inunction is to be preferred, except in cases where the absorptive powers of the skin are impaired or where a more rapid entrance into the circulation is desired. Other uses in the form of bougies or suppositories will suggest themselves, but time does not permit of their discussion here.

The metal has as yet never been found in the urine or kidneys. The feces often assume a brownish, black color after its use, which suggests the liver as a possible organ of elimination. No matter what the effect upon the condition for which it is administered, silver in the largest quantities has never been known to cause any ill results and no case of argyria has been reported, even where treatment has been prolonged for months.

Now having considered silver as a remedy for sepsis, let us look at its indications and limitations. We must not be unreasonable in our expectations, and it is absurd for any one to look for the absorption of a large collection of pus or for the restoration of a gangrenous appendix by the application of any remedy. The principals of aseptic surgery should always be carried out where they are indicated. Nor is it reasonable to expect too much in cases where the toxins by their virulence or prolonged action, have produced too great a degeneration in the heart or kidneys. It is perfectly possible for a septic condition to improve and the patient to die from the irreparable damage done to these organs. This is true in diphtheria in the use of antitoxin, as those who have observed will testify.

The clearest indications for collargolum are beginning inflammations due to the pyogens. Under this we include cellulitis before formation of pus has occurred, both in the pelvis and other situations; erysipelas and puerperal sepsis; inflammation of operative wounds; ostitis and many other conditions of inflammation that will suggest themselves. The many reports under the above indications, in the use of silver, have been very gratifying and so successful indeed has it been that failure in clear indications leads one to suspect faulty technique or insufficient dosage.

In literature we find reports on the use of silver in every form of sepsis and infectious disease, but in the latter there seems to be no uniformity of result. True it is that Credé seems in his clinic to get results in other forms of infection. I shall not take your time to cite the eminent authorities who believe in silver for sepsis, nor to mention their reports, but shall refer you to the literature appearing from time to time on the subject. In accepting cases we ought to always bear in mind that sepsis in its course is fickle, spontaneous resolution often taking place where no especial treatment has been instituted, and it is only after a large number of cases have been reported and compared that we may feel that the indications and effects of silver are definitely defined.

The experience that competent observers have had with it, justify our employment of the remedy, as it certainly, without question, is the best we have at the present day to combat sepsis. It being absolutely nonpoisonous and followed by no ill-effect, is an advantage which should encourage and promote its use. My own experience, I confess, has not been large in the use of silver and the citation of one case in which the indications for its use were clear and the result of treatment seemed dependent on its use will be sufficient:

Mrs. D., aged 26; always a well woman and the mother of two children. Finding herself pregnant between three and four months she undertook to bring on abortion by the injection of water on several different occasions, into the uterus. At the end of a week the pain and hemorrhage were so severe that a neighboring physician was called, who after removing the fetus that presented at the os, left her. A day or so following, I was called and with the placental forceps removed the placenta that I found in the os. Uterus was washed out. A few days later a sharp hemorrhage occurred, but ceased spontaneously, and curettage was advised and refused. Patient was not seen again for ten days, when I was called and found her in bed, and I was told that for past week she had been about, and for three days had had pain in right side, gradually getting worse. Temperature, 101°; pulse, 100; examination showed beginning cellulitis of broad ligament; exudate easily felt. At the end of three days the process had extended in front and behind the uterus and the mass in broad ligament was much larger; urination and defecation very painful; lower abdomen tender to the slightest touch; vomiting present and morphine required several times daily by hypodermic. Temperature, 103° and pulse, 120. No chill at any time. A dram of unguentum Credé was rubbed well in at night; this was repeated the following morning and during that day one hypodermic only was required; that evening fround her quite comfortable. Temperature, 100°; pulse, 110. Another dram of the ointment was used and the following morning I found my patient very comfortable and a eighth of morphine only was required during that day. Evening temperature, 99.2-5; pulse, 96. Examination showed mass still present, but much less tender; abdomen also improved. The improvement from this time was rapid; rises of temperature were transient; the ointment was continued once daily for several days. It required several weeks for entire absorption of the exudate, but the improvement was uninterrupted.

The only treatment used with the silver was hot douches, which I am sure had but little effect, because of the pain it was not possible to get the best results from them. This result too coincides with others in similar cases, and I believe that I am justified in attributing the rapid improvement to the use of silver. The other cases in which I used it were pus cases, which after the pus was evacuated followed the ordinary course to recovery. One other was a case of acute bulbar paralysis in which the patient died in 48 hours after the first symptoms appeared.

I have tried to show what collargolum is, the method of its application and the indications for its use. Perhaps many have employed it and met with disappointment. I can only urge further trial, using it as early in cases as possible. We do not always find antitoxin successful, but the unsuccessful cases are the late ones. Give unguentum Credé the same chance as antitoxin and use it in sufficient quantities, and there is no question but what in properly selected cases results will be obtained.

### DISCUSSION.

Dr. Grover Wende said he had tried it on 25 to 50 cases of impetigo contagiosa, where it was directly indicated. In these lesions there is a mixture of staphylococci and streptococci. The lesion being superficial the whole area can be covered with the application. He had also tried it in pustular eczema, the latest stages of which both the pyogens are found. He got no results. On the other hand, the old remedy—ammoniated mercury—will produce decided results in impetigo in 24 to 48 hours. Dr. Wende advanced the suggestion that the silver acted only upon the toxins and not upon the germs themselves. This will account for its failure in the foregoing where no constitutional disturbances are ever present.

Dr. A. G. BENNETT said he had tried it in one case of rather a rare disease—descemetitis—an inflammation of the whole uveal tract, that is taking in the choroid, ciliary body and iris. The iris is always inflamed generally with adhesions; there are always deposits, on the interior surface of the cornea. Of all diseases that he has ever treated, with one exception, this is the most obstinate. This is the only case of that disease that was ever cured. He had had seven or eight that never effected a cure until he used Credé's ointment on this one. It was due to systemic gonorrheal infection. And the case was as bad as he had ever seen. Inunctions were given once a day, a dram each time. He also reported success in gonorrheal bubo, which under inunctions rapidly subsided.

Dr. W. E. DIGNEN said he had used it on one case of sepsis following miscarriage. The results were good from some cause or other.

Dr. GEO. F. COTT said he had seen it used in one case. He had been called in consultation in a case of apparent middle-ear disease. There had been sore throat for some days, then pain in the ear until rupture of the drum-head with the escape of serum. This did not remedy matters much for the pain appeared in the external canal, which the physician had punctured the day before, finding purulent material and blood. The canal was excessively swollen and could not be examined. The skin, the periosteum and the external canal were freely incised with the escape of considerable blood. The next day the temperature was 104°. The day following the doctor saw her and suggested erysipelas which had begun in the throat, invaded the middle-ear and then extended in the external canal. A culture revealed the presence of streptococci in the secretions. Credé's ointment, one ounce daily, was used in conjunction with antistreptococcic serum. Result: uninterrupted recovery.

Dr. KING—To the question on the use of silver in gonorrhea I would say that the reports of the use of Credé's preparation in gonorrhea were conflicting. Credé told me personally of a large number of cases treated by an unction of which a fair proportion treated in this manner showed marked improvement and speedy cure; other cases required additional treatment. In gonorrheal rheumatism he had had better success and quite uniform results.

In regard to Dr. Wende's experience I have never seen it used as a simple application. Credé never uses it in that way, always using the systemic treatment by inunction, never simply by applying the ointment. In answer to Dr. Gibson's question as to the mode of application, I would state that it was a very important part of the procedure. Its proper application sometimes means success, where otherwise you would have failure. Credé says that a large number of the reported failures are undoubtedly due to the faulty methods of application. First be sure that the patient's skin is in such condition that absorption may occur; prepare it by scrubbing and softening, then apply the ointment by friction, continuing twenty to thirty minutes, or even longer, in order that the whole amount may be taken up by the skin. The usual locations for inunctions are chosen. In regard to the effect of repeated applications on the same spot he could not speak, having never seen it done.

In cases where the silver has not been absorbed by the skin, Credé resorts to the intravenous methods; but unless contraindicated, he always uses inunctions.

Doses depend altogether on results. Any amount may be used without fear of ill effects, either internally or by inunction. There never has been a report indicating that there had been any untoward symptoms, as an ounce a day has been used without any bad effects. The usual dose is anywhere from 15 to 45 grains to the inunction. It may be increased either in amount or frequency of application. Collargolum is used internally in aqueous solution. All solutions are much more stable if albumin be added. Albumin requires administration by mouth, for then the gastric juice does not cause precipitation. Solutions should be freshly made and protected from the light. By the addition of albumin, keeping in the dark and in closely stopped bottles, a solution can be kept for several weeks. The better plan is to keep it in powder and make it fresh. The drug may be administered at intervals of four, six or eight hours, always remembering it has no poisonous effects.

Credé exhibited cultures of various organisms in which he had placed bits of silver wire. For an area around each piece of wire, about an eighth of an inch, bacteria failed to grow, while in all other locations they multiplied in great abundance. In other experiments, he showed the incubation of germ growth by the action of these preparations.

One of Credé's favorite treatments is by placing a pill or bougie in a sinus or wound and leaving it. The same thing is done in sepsis of the uterus. A pill or bougie is left two or three days, the gauze being packed against the surface.

Dr. King had never seen the preparation used in syphilis.

